# Analysis of microglial BDNF function and expression in the motor cortex

**DOI:** 10.3389/fncel.2022.961276

**Published:** 2022-12-23

**Authors:** Diana Honey, Erin Wosnitzka, Eric Klann, Laetitia Weinhard

**Affiliations:** ^1^NYU Grossman School of Medicine, New York, NY, United States; ^2^Center for Neural Science, New York University, New York, NY, United States; ^3^Department of Fundamental Neurosciences, UNIL, Lausanne, Switzerland; ^4^Cardiff School of Biosciences, Cardiff University, Wales, United Kingdom

**Keywords:** microglia, BDNF, plasticity, spine, calcium

## Abstract

Brain-derived neurotrophic factor (BDNF) is a neurotrophin that regulates several aspects of brain function. Although numerous studies have demonstrated the expression and function of BDNF in neurons, its expression in microglia remains controversial. Using a combination of genetic tools and fluorescence imaging, we analyzed BDNF expression patterns and investigated the effect of microglial *Bdnf* deletion on neuronal activity, early-stage spine formation, and microglia-neuron attraction in the motor cortex. We did not detect BDNF expression in microglia at the transcriptional or translational level, in physiological or pathological conditions, and none of the assessed neuronal functions were found to be affected in conditional *Bdnf* knockout mice. Our results suggest that microglia do not express BDNF in sufficient amounts to modulate neuronal function.

## Introduction

BDNF is a member of the neurotrophin family first described to promote neuronal survival (Barde et al., 1982). Additional roles for BDNF in modulating neuronal activity and synaptic plasticity have since been extensively documented (Chao, 2003). In particular, BDNF was reported to facilitate long-term potentiation (Figurov et al., 1996; Kang et al., 1997) and spine enlargement (Rex et al., 2007). Furthermore, BDNF post-translational maturation was shown to be regulated by physical exercise *via* the tissue plasminogen activator (tPA; Pang et al., 2004; Ding et al., 2011; Leckie et al., 2014).

Microglia are a component of the innate immune system and are increasingly recognized to be important regulators of neuronal function. In particular, microglia were recently shown to modulate neuronal activity (Badimon et al., 2020; Cserép et al., 2020; Merlini et al., 2021) and mediate structural plasticity *via* the induction of postsynaptic protrusions (Miyamoto et al., 2016; Weinhard et al., 2018). BDNF expression in microglia was originally reported through *in vitro* experiments (Elkabes et al., 1996; Batchelor et al., 1999) and was suggested to be upregulated by ATP *via* P2X4R (Coull et al., 2005; Trang et al., 2009; Malcangio, 2017) and after LPS stimulation (Prowse and Hayley, 2021). In the spinal cord, microglia were proposed to mediate allodynia after nerve injury *via* the release of BDNF (Coull et al., 2005). In the brain, conditional knockout of microglial *Bdnf* impairs training-induced spine formation in the motor cortex (Parkhurst et al., 2013), and blocks nerve injury-induced neuronal hyperactivity in the somatosensory cortex (Huang et al., 2021). Despite this body of evidence suggesting an important biological role for microglial BDNF in regulating neuronal function, transcriptomic analyses from cerebral and spinal microglia consistently report very low levels of BDNF expression (Bennett et al., 2016; Denk et al., 2016; Ayata et al., 2018; Kang et al., 2018). Therefore, it remains unclear whether microglia produce BDNF in sufficient quantities *in vivo* to modulate neuronal function.

In this study, we evaluated the role of microglial BDNF in modulating protrusion formation, training-evoked neuronal activity, and microglia-neuron interaction in the motor cortex under physiological conditions. We combined genetic tools with two-photon *in vivo* imaging to visualize neurons and microglia while specifically deleting *Bdnf* from microglia. We observed no effect of microglial *Bdnf* deletion on neuronal activity, protrusion formation, or microglia-neuron contacts. Finally, we used a *Bdnf-p2a-Gfp* and a *Bdnf::2a-cre* reporter system to characterize BDNF expression patterns in the brain. Although the vast majority of brain cells expressed the BDNF reporter, we did not observe any homeostatic expression in microglia in resting conditions, after training, or in inflammatory conditions. Taken together, our results suggest that microglia do not express physiologically-relevant levels of BDNF to participate in the regulation of neuronal function in the motor cortex.

## Materials and Methods

### Experimental animals

All experimental protocols were conducted according to the National Institutes of Health (NIH) guidelines for animal research and approved by the Institutional Animal Care and Use Committee (IACUC) at New York University Medical Center (IACUC protocol: 160905).

To analyze the effect of microglial BDNF on protrusion formation, *Thy1*-YFP-H; *Cx3cr1*^creER/+^; *R26*^LSL-tdTomato^; *Bdnf*^f/f^ quadruple transgenic mice were generated by crossing *Thy1*-YFP-H (Jackson Laboratory stock 003782) with *Cx3cr1*^creER-YFP^ (Jackson Laboratory stock 021160), *Rosa26*^CAG-LoxP-Stop-LoxP-tdTomato-WPRE^ (Jackson Laboratory stock 007905) and *Bdnf*-floxed mice (Jackson Laboratory stock 033689). To analyze the effect of microglial BDNF on neuronal calcium activity, *Thy1*-GCaMP6s; *Cx3cr1*^creER/+^; *R26*^LSL-tdTomato/+^; *Bdnf*^f/f^ quadruple transgenic mice were generated by crossing the aforementioned mice with *Thy1*-GCaMP6s (Jackson Laboratory stock 024275). Cre-mediated recombination was induced by two injections of 98% Z- isomers hydroxy-tamoxifen diluted in corn oil at 10 mg/ml (Sigma, 1 mg injected per 20 g of mouse weight) at Postnatal day 15 (P15) and P21. Recombination was confirmed by the expression of tdTomato in virtually all YFP+ microglia. *Thy1*-GCaMP6s (or *Thy1*-YFP); *Cx3cr1*^creER/+^; *R26*^LSL-tdTomato^; *Bdnf*^f/f^ were compared with *Thy1*-GCaMP6s (or *Thy1*-YFP); *Cx3cr1*^creER/+^; *R26*^LSL-tdTomato/+^; *Bdnf*^+/+^ littermates, blind to genotype.

For BDNF expression pattern analysis, *Bdnf*::*2a-cre*/+; *R26*^LSL-tdTomato/+^; *Cx3cr1*^GFP/+^ mice were generated by crossing *Bdnf::2a-cre* mice (Jackson Laboratory stock 030189) with *Rosa26*^CAG-LoxP-Stop-LoxP-tdTomato-WPRE^ and *Cx3cr1*^GFP^ (Jackson Laboratory stock 005582). P60 and P140 mice were perfused transcardially under anesthesia using 4% paraformaldehyde (PFA) and brains were removed and post-fixed in 4% PFA overnight at 4°C. Coronal sections were cut on a vibratom at 50 μm (Leica Microsystems, Wetzlar, Germany). ATP treatment was performed a week before perfusion, by injecting 0.5 μl of 500 μM ATP in the motor cortex through a hole drilled in the skull at 1 mm from Bregma, and 1.2 mm lateral from the midline. LPS treatment was performed 2 days before perfusion by injecting 0.5 μl of 100 μM LPS.

### Immunohistochemistry

*Bdnf-P2a-Gfp* mice were killed at P90 by pentobarbital injections transcardially perfused with ice-cold PBS and 4% PFA. Their brains were removed and postfixed at room temperature for 4 h before cryoprotecting in 30% w/v sucrose solution at 4°C overnight. The following day, brains were embedded in OCT (optimal cutting temperature) compound and sectioned at 40 μm using a cryostat. Sections were blocked in blocking solution (3% donkey serum and 4% BSA in PBS-T) for 1 h before incubating overnight with chicken anti-GFP (Abcam 13970, 1:1,000) and rabbit anti-Tmem119 (Abcam 209064, 1:500) or rabbit anti-NeuN (Abcam 177487). Sections were then washed three times for 10 min with PBS-T before incubating with Alexa Fluor 555 anti-rabbit IgG and Alexa Fluor 488 anti-chicken IgY secondary antibodies (1:500; Thermo Fisher Scientific) for 1 h at room temperature. After a final wash in PBS-T for 10 min, sections were incubated with DAPI diluted in PBS (1:4,000) for 20 min and mounted onto precoated polylysine slides (VWR) with Dako fluorescence mounting media. Two sections from two animals were analyzed.

### Surgery and *in vivo* imaging

Two-photon imaging was carried out in awake, head-restrained mice through a thinned-skull window. For mounting the head holder, mice were deeply anesthetized with an intraperitoneal injection of ketamine (100 mg) and xylazine (10 mg). The head was shaved and the skull surface exposed with a midline scalp incision. The periosteum tissue over the skull surface was removed without damaging the temporal and occipital muscles. A head-holding device made using two parallel metal bars was attached to the skull to help restrain the animal and reduce motion-induced artifacts during imaging. The holder was mounted on top of the skull with dental acrylic cement and cyanoacrylate glue. Precaution was taken to leave exposed the skull region corresponding to the motor cortex (1 mm from bregma and 1.2 mm lateral from the midline). The completed cranial window was covered with silicone elastomer (World Precision Instruments) and the animals were returned to their home cage to recover. Imaging experiments started >24 h after window implantation. Mice were habituated for 10 min to the treadmill-imaging apparatus to minimize potential stress effects of head restraining, motor training, and imaging. Before imaging, the silicone was removed and the skull was thinned by carefully scraping the cranial surface with a microsurgical blade down to 20 μm in thickness. Mice were then head restrained under the microscope, which sits on top of the custom-built free-floating treadmill, and the objective immersed in ACSF-filled head mount. Two-photon imaging was performed with a Bruker two-photon system equipped with a 60× objective (NA 1.05) and a Ti:Sapphire laser (MaiTaiDeepSee, Spectra Physics) tuned to 965 nm. The average laser power on the L1 cortex tissue was ~30 mW.

For protrusion formation and microglia dynamics analyses, z-stacks were acquired in layer 1 of the motor cortex every 5 min over 1 h before training, and over 1 h after training at 0.15 μm/pixel. For calcium activity experiments, images at a single z-plane were acquired at a frame rate of ~2 Hz over 50 s at 0.45 μm/pixel. All images were registered using ImageJ to correct for motion artifacts.

### Treadmill training

A custom-built free-floating treadmill (100 cm × 60 cm × 44 cm) was used for motor training under a two-photon microscope. This free-floating treadmill allowed head-fixed mice to move their forelimbs freely to perform motor running tasks (forward or backward). To minimize motion artifacts during imaging, the treadmill was constructed so that all the moving parts (motor, belt, and drive shaft) were isolated from the microscope stage and the supporting air-table. Animals were positioned *via* a custom-made head-holder device that allowed them to be held in place during motor training. The treadmill motor was driven by a DC power supply. At the onset of the trial, the motor was turned on and the belt speed gradually increased from 0 cm/s to 4 cm/s within ~3 s, and the speed of 4 cm/s was maintained for the rest of the trial. For analysis of protrusion formation, mice were trained for three trials (8 min running and 2 min resting). For calcium activity analysis, mice underwent two trials of 30 s forward running, and two trials of 30 s backward running. For induction of BDNF expression after motor training, a wheel was installed in the animals’ home cage for 3 days, and the mice were placed in closed wheel 4 h per day under experimenter control to ensure motor activity.

### Protrusion analysis

For protrusion analysis, six animal pairs were analyzed at P60. For each animal, seven dendritic segments of 50 μm were selected from *Thy1*-YFP neurons based on their brightness and position 10–50 mm under the pia. The number of new protrusions (filopodia or spine) formed over an hour was manually annotated.

### Microglia motility analysis

Microglia motility was analyzed from three littermate pairs at P60. Putative microglia contacts per 50 μm dendritic segment (selected as described above) were counted for each timepoint, as well as the number of new contacts and contact loss between timepoints (5 min lapse). The interaction index was calculated at each timepoint by summing the addition and loss of new and lost microglia-dendrite contacts, divided by the absolute number of contacts per timepoint. Intensities were thresholded as follows: positive microglia were used as a reference and their measured intensity brought up to maximal value (255 of 0–255 8-bits scale). Lower threshold values were defined as residual signal measured in the center of cross-sectioned vessels +30%, and brought to 0. Contacts were defined as colocalization of >3 consecutive pixels (>0.45 μm) in green and red channels.

### Calcium activity analysis

For calcium activity analysis, images from four littermate pairs were analyzed at P60 using ImageJ. Lateral movements were corrected using tdTomato-positive microglia as a reference. Vertical movements were controlled using microglia as a reference, and found to be infrequent due to the flexible belt design and custom stage. Before analysis, microglial signal was subtracted in both channels. Regions of interest (ROIs) of 20 × 20 pixels was used for quantification of fluorescence intensity (F) on dendrites identified for being active in at least one training session. The ΔF/F_0_ value was calculated as ΔF/F_0_ = (F − F_0_)/F_0_ where F_0_ is the baseline fluorescence signal measured as the average of the 10 lowest measured F values in one session. Dendritic calcium spikes were defined as >60% ΔF/F_0_ and >60% of the highest measured ΔF/F_0_ for each dendrite. Most calcium spikes ranged from 70% to 300% signal increase.

### BDNF expression pattern analysis

For BDNF expression pattern analysis, mice were perfused at P60 and P140. Two slices from two animals per condition were analyzed. The number of DAPI +, tdTomato+, and GFP+ cells were counted in ROIs set at 214 × 70 × 25 μm (374,000 μm^3^). Layer 1 analysis was set at 5–75 μm under the pia, Layer 2/3 at 100–170 μm, and Layer 5 at 350–420 μm. Only cells with at least 50% of their nuclei contained within the examined volume were counted. Intensities were thresholded as follows: the brightest tdTomato- or GFP- positive cells were used as a reference and brought up to the maximal value (255 of 0–255 8-bits scale). Lower threshold values were defined as residual signal measured in the center of cross-sectioned vessels +30%, and brought to 0. Colocalization analysis was performed for each DAPI-positive cell by examining tdTomato and GFP signal in the center of the nuclei.

### Statistical analysis

All statistical analyses were performed using Graphpad. For each dataset, Gaussian distribution was assessed using Shapiro-Wilk normality test. Normal datasets following a Gaussian distribution were compared using unpaired or paired *t*-tests. Datasets following a non-Gaussian distribution were compared using the non-parametric Mann-Whitney U test. Datasets with two variables were compared using a two-way ANOVA.

## Results

### Deletion of *Bdnf* in microglia does not alter protrusion formation in the motor cortex

**Figure 1:**
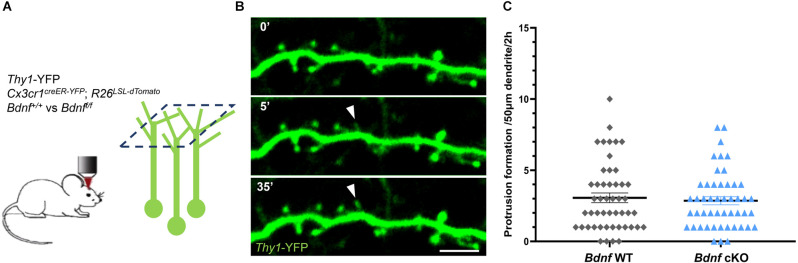
Deletion of microglial *Bdnf* does not affect protrusion formation. **(A)** Paradigm to assess protrusion formation using two-photon *in vivo* imaging of *Thy1-YFP* neurons in L1 of the motor cortex at P60. *Thy1*-YFP; *Cx3cr1*^creER-YFP/+^; *R26*^LSL-tdTomato/+^; *Bdnf*^+/+^ (named *Bdnf* WT) mice and their *Thy1*-YFP; *Cx3cr1*^creER-YFP/+^; *R26*^LSL-tdTomato/+^; *Bdnf*^f/f^ littermates (*Bdnf* cKO) were imaged over 2 h at one image every 5 min. **(B)** Representative time sequence of protrusion formation (full arrowhead). **(C)** No difference in protrusion formation was observed between *Bdnf* WT and *Bdnf* cKO mice littermates at P60 (*n* = 49 dendrites from seven animals in each group, *p* = 0.91, Mann-Whitney test). Scale bar: 5 μm.

We first assessed whether microglial BDNF mediates structural plasticity by examining the formation of postsynaptic protrusions in a conditional *Bdnf* knockout model. We crossed *Thy1*-YFP mice that express YFP in a subset of excitatory pyramidal neurons in Layer 5 (L5) projecting apical tuft dendrites to Layer 1 (L1), with *Cx3cr1*^creER-YFP^; *R26*^LSL-tdTomato^ and *Bdnf*-floxed mice to specifically delete *Bdnf* from microglia while inducing tdTomato expression upon tamoxifen injection. To assess the efficiency of tamoxifen-induced recombination, we analyzed tdTomato reporter expression in microglia. We found that the vast majority of YFP-labeled microglia co-expressed tdTomato (39/40 cells analyzed from eight animals; Supplementary Figure 1), demonstrating that tamoxifen effectively induced nuclear translocation of the cre recombinase and loxP sites recombination. Next, we performed two-photon *in vivo* imaging of YFP-labeled dendrites in L1 of the motor cortex in awake mice at postnatal day 60 (P60), and compared the dynamics of spine formation between *Bdnf* wild-type (*Bdnf* WT) and conditional *Bdnf* knockout (*Bdnf* cKO) mice (Figure 1A). We frequently observed the formation of transient protrusions (Figure 1B), however there were no difference in protrusion formation between *Bdnf* WT and *Bdnf* cKO mice over 2 h (Figure 1C, 3.1 ± 0.3 and 2.9 ± 0.3 protrusions per 50 μm in 2 h respectively). We also observed that motor training did not affect protrusion formation immediately after the session (Supplementary Figure 2), confirming previous studies suggesting a protracted effect of neuronal activity on spine formation (Yang et al., 2014). These results indicate that microglial BDNF does not significantly regulate spine formation.

### Deletion of *Bdnf* in microglia does not alter training-evoked neuronal activity in the motor cortex

**Figure 2:**
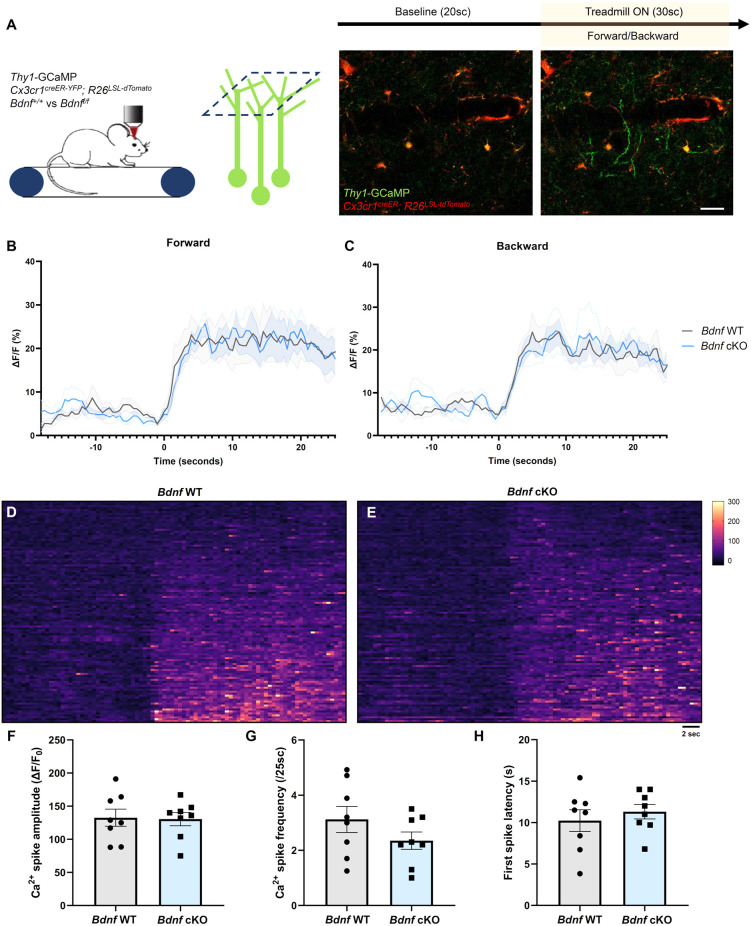
Deletion of microglial *Bdnf* does not affect dendritic activity. **(A)** Paradigm to assess training-induced dendritic activity using two-photon imaging of *Thy1-GCaMP6s* in L1 of the motor cortex, and representative example of dendritic activity after the treadmill was turned on. **(B,C)** Average Ca^2+^ activity in the neuropil of *Thy1*-GCaMP6s; *Cx3cr1*^creER-YFP/+^; *R26*^LSL-tdTomato/+^; *Bdnf*^+/+^ (*Bdnf* WT) mice and *Thy1*-GCaMP6s; *Cx3cr1*^creER-YFP/+^; *R26*^LSL-tdTomato/+^; *Bdnf*^f/f^ littermates (*Bdnf* cKO). No difference was observed between genotypes during **(B)** forward or **(C)** backward running (eight training sessions from four animals in each group). **(D,E)** Heatmaps of dendritic Ca^2+^ activity (112 dendrites measured from two sessions, from four animals in each group). **(F–H)** Dendritic Ca^2+^ spike analysis (*n* = 8 sessions from four animals in each group) showing **(F)** no significant difference in spike amplitude (*p* = 0.9, unpaired *t*-test), **(G)** no significant difference in spike frequency (*p* = 0.2, unpaired *t*-test) and **(H)** no significant difference in first spike latency from training onset (*p* = 0.5, unpaired *t*-test). Scale bar: 10 μm.

To evaluate whether microglial BDNF modulates neuronal activity, we crossed *Thy1*-GCaMP6s mice that express GCaMP in a subset of L5 excitatory pyramidal neurons with apical tuft dendrites in L1, with *Cx3cr1*^creER-YFP^; *R26*^LSL-tdTomato^ and *Bdnf*-floxed mice to specifically delete *Bdnf* from microglia upon tamoxifen injection. We performed transcranial two-photon *in vivo* imaging in the motor cortex of awake mice at P60 and characterized training-induced neuronal calcium activity during forward and backward running (Figure 2A). L1 was imaged for 20 s before the treadmill was turned on for 30 s, inducing a robust increase in dendritic calcium activity (Figure 2A). First, we measured calcium activity in the neuropil, which comprises presynaptic boutons and dendrites. No difference in neuropil activity was observed between *Bdnf* WT and *Bdnf* cKO littermates during forward or backward running (Figures 2B,C). To gain more resolution, we then focused our analyses on dendrites and measured the calcium signal in dendritic segments displaying a spike during at least one of the two forward training sessions (Figures 2D,E). We did not observe a significant difference in calcium spike amplitude, frequency, or onset latency between genotypes (Figures 2F–H), although we did notice a trend for lower spike frequency in *Bdnf* cKO mice (Figure 2G). These results suggest that microglial BDNF does not modulate training-evoked neuronal activity in the motor cortex under physiological conditions.

### Microglial BDNF does not affect microglia-neuron contacts

**Figure 3:**
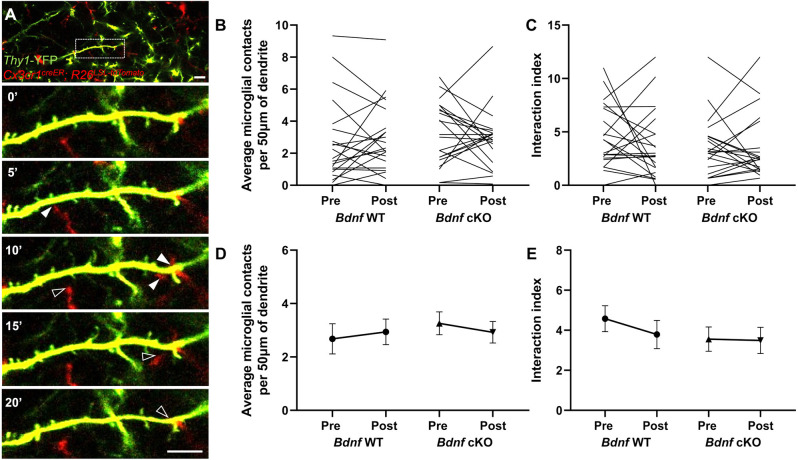
Deletion of microglial *Bdnf* does not affect microglia-neuron contacts. **(A)** Representative time sequence of microglia-dendrite contacts, displaying new contacts (full arrowhead) and contact loss (empty arrowhead). **(B,D)** Number of microglia-dendrite contacts per timepoint pre- and post-training, plotted **(B)** per dendrite and **(D)** as average. Although most dendrites display a shift in the number of contacts after training **(B)**, there was no difference in the average **(D)** pre/post training (21 dendrites analyzed from three animals in each group, *p* = 0.94, two-way ANOVA), or between genotypes (*p* = 0.55, two-way ANOVA). **(C,E)** Calculation of the interaction index (sum of new and lost contacts divided by the number of contacts per timepoint) revealed no difference in the dynamics of microglia-dendrite contacts pre/post (21 dendrites analyzed from three animals in each group, *p* = 0.68, two-way ANOVA), or between genotypes (*p* = 0.52, two-way ANOVA). Scale bar: 10 μm.

To evaluate whether BDNF plays a role in microglia attraction towards neurons, we assessed the dynamics of putative contacts between microglia and neurons. We performed two-photon *in vivo* imaging before and after motor training in L1 of the motor cortex in *Thy1*-YFP; *Cx3cr1*^creER-YFP/+^; *R26*^LSL-tdTomato/+^; *Bdnf*^f/f^ mice. We observed that tdTomato-labeled microglia made frequent contacts with YFP-labeled dendrites over 1 h (Figure 3A). We quantified the absolute number of microglial contacts per dendritic segment per timepoint (Figures 3B,D) and evaluated the dynamics of these contacts by calculating an interaction index summing the new and lost contacts per timepoint, normalized by the total number of contacts per segment (Figures 3C,E). For the majority of the dendrites analyzed, training induced shifts in the number and dynamics of microglia-dendrite contacts that were comparable between genotypes (Figures 3B,C). Collectively, we found no significant differences in the number (Figure 3D) or dynamics (Figure 3E) of microglia-neuron contacts before/after training or between genotypes. These results suggest that microglial BDNF does not modulate microglia-neuron interactions.

### Microglia do not express BDNF at the translational level

**Figure 4:**
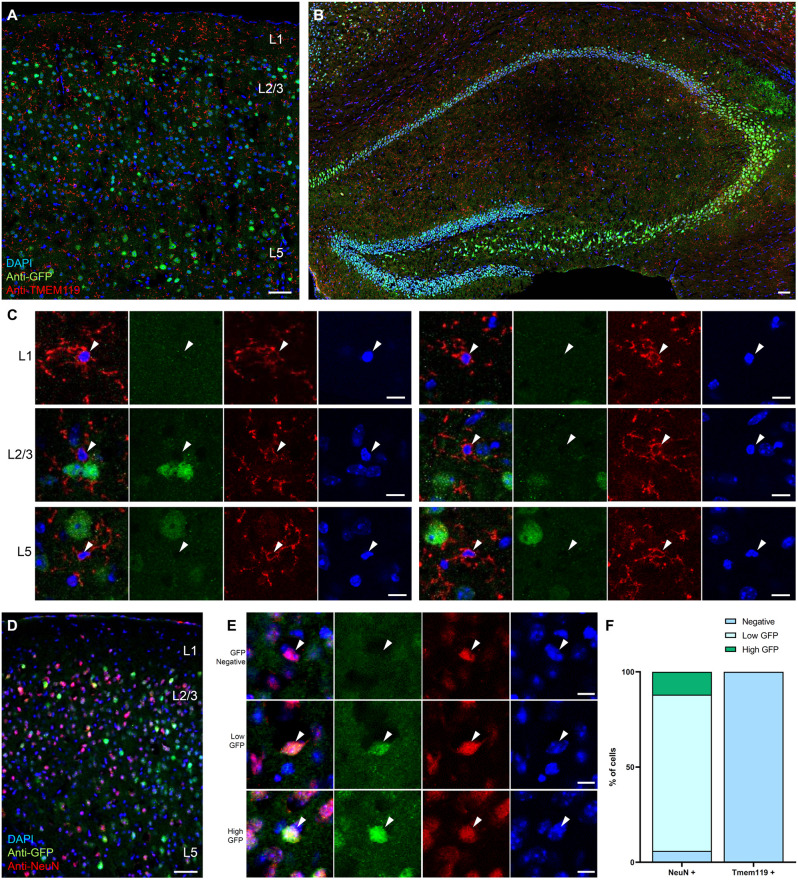
Microglia do not express the reporter for BDNF translation BDNF-P2A-GFP. Low magnification images of the *Bdnf-P2a-Gfp* reporter mouse line immunostained against the microglial marker TMEM119 (red), in **(A)** the motor cortex and in **(B)** the hippocampus. **(C)** Representative high magnification images of TMEM119 expressing microglia (red) from L1, L2/3, and L5 showing the absence of GFP signal. **(D)** Low magnification image of the motor cortex from a *Bdnf-P2a-Gfp* mouse cortical section immunostained against the neuronal marker NeuN (red). **(E)** Representative high magnification images of NeuN expressing neurons (red) showing differential levels of *Bdnf* mRNA translation (GFP). **(F)** Quantification of the percentage of neurons and microglia expressing BDNF-P2A-GFP. Scale bar: **(A,B,D)**: 50 μm, **(C,E)**: 10 μm.

Given that none of the neuronal functions tested were affected by the conditional knockout of *Bdnf* in microglia, and very low levels of microglial *Bdnf* mRNA were reported in transcriptomic datasets (Kang et al., 2018), we next investigated BDNF expression in microglia. Because BDNF is difficult to detect with specificity using commercially available reagents, we developed several transgenic reporter strategies. To visualize BDNF expression at the translational level, we took advantage of a recently generated reporter mouse line in which the endogenous *Bdnf* gene has been replaced by a bicistronic *Bdnf-Gfp* separated by a P2A sequence, thereby allowing GFP expression upon *Bdnf* mRNA translation (Wosnitzka et al., 2020). At P90, differential levels of expression were observed in cell bodies throughout the cortex and hippocampus (Figures 4A–E), as previously described (Wosnitzka et al., 2020). We performed immunohistochemistry against TMEM119, a transmembrane protein and a specific marker for microglia (Bennett et al., 2016), and analyzed the colocalization of TMEM119-positive microglia with GFP-positive cells (Figures 4A–C). Of the 152 microglia analyzed, none expressed GFP (*n* = 102 cells in the motor cortex and *n* = 50 cells in the hippocampus), indicating that microglia do not translate *Bdnf* mRNA. In contrast, immunohistostaining against the neuronal marker NeuN revealed that 94% of neurons express GFP in the motor cortex (Figures 4D–F, *n* = 94/100 cells analyzed), among which 15% displayed expression above the average of all measured cells (*n* = 12/100 cells). These results suggest that, unlike neurons, microglia do not translate *Bdnf* mRNA in the motor cortex.

### Microglia do not express BDNF at the transcriptional level

**Figure 5:**
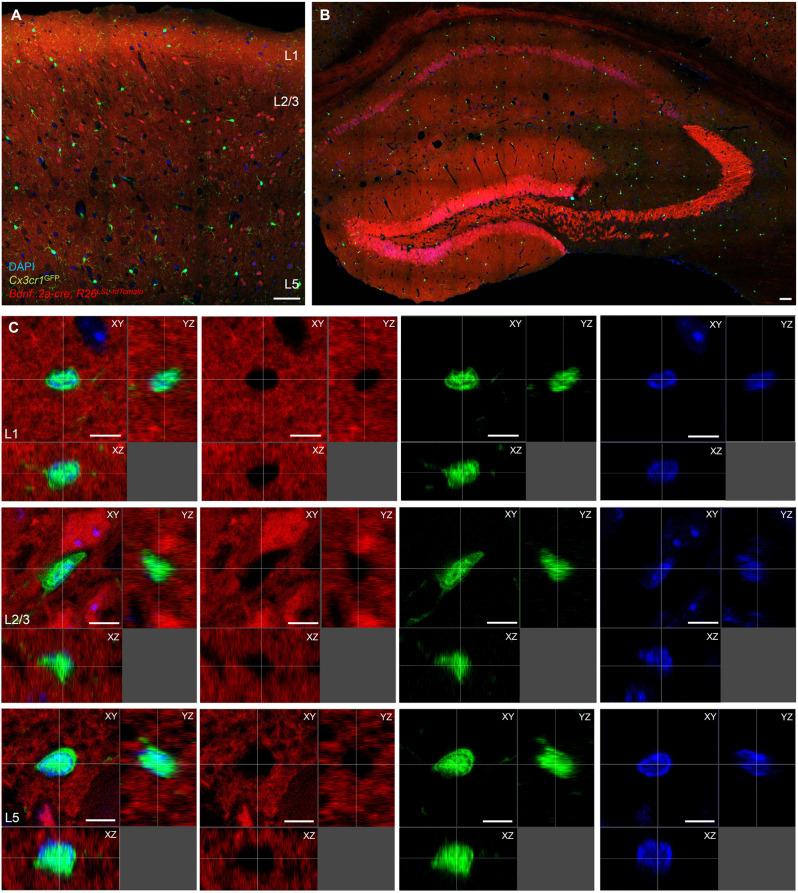
Microglia do not express the reporter for BDNF transcription *Bdnf::2a-cre*; *R26*^LSL-tdTomato^. **(A)** Low magnification image of a coronal section from the *Bdnf::2a-cre/+*; *R26*^LSL-tdTomato/+^; *Cx3cr1*^GFP/+^ mouse reporter line at P60. **(B)** Low magnification image of a hippocampal section. **(C)** Representative three-dimensional images of GFP-labeled microglia at high magnification showing the absence of tdTomato signal within GFP-labeled microglia. Scale bar: **(A,B)**: 50 μm, **(C)**: 10 μm.

Next, we investigated the expression of BDNF by microglia at the transcriptional level by crossing a constitutive *Bdnf::2a-cre* mouse line with the tdTomato reporter *R26*^LSL-tdTomato^ and the microglial reporter *Cx3cr1*^GFP^. At P60, tdTomato expression was detected throughout the brain, with strong labeling of the cortex (Figure 5A) and hippocampus (Figure 5B). Based on the size and shape of the tdTomato-labeled cell bodies and processes, and the overwhelming presence of labeling in neuronal layers such as layer 2/3, layer 5, and *stratum pyramidale* (Figures 5A,B), the majority of tdTomato positive cells appeared to be neurons. We occasionally observed stellar, highly arborized tdTomato-positive cells, suggestive of astrocytic morphology (Supplementary Figure 3A), as well as vessel-lining tdTomato-positive cells (Supplementary Figure 3B).

We performed co-localization analysis of tdTomato and GFP-positive cells at P60 in layer 1 (L1), layer 2/3 (L2/3), and layer 5 (L5) of the motor cortex (Figure 5C), as well as in the *Stratum Oriens* (SO), *Stratum Pyramidale* (SP), and *Stratum Radiatum* (SR) of the hippocampus (Supplementary Figure 4). As previously reported (Jung et al., 2000), the *Cx3cr1*^GFP^ reporter mostly labeled highly branched microglia (Figures 5A,B). In rare instances, we observed vessel-associated cells in the SO and SR (Supplementary Figure 3C), and neurons in the SP (Supplementary Figure 3D) that faintly expressed GFP and tdTomato, suggesting that some non-microglial cells co-express CX3CR1 and BDNF. However, we did not observe any GFP-labeled microglia expressing tdTomato in any of the regions analyzed (*n* = 98 cells in the motor cortex, and *n* = 36 cells in the hippocampus, from two animals at P60), as three-dimensional representations demonstrated clear exclusion of tdTomato signal within GFP-positive microglial cell bodies (Figure 5C). In contrast, the same *R26*^LSL-tdTomato^ reporter crossed with the microglia-specific *Tmem119*^creER^ mouse line yielded strong tdTomato expression in microglia that filled cell bodies and processes (Supplementary Figure 5). Our results again indicate that microglia do not express detectable amounts of BDNF under physiological conditions.

### Motor training or pro-inflammatory stimuli do not induce BDNF expression in microglia

**Figure 6:**
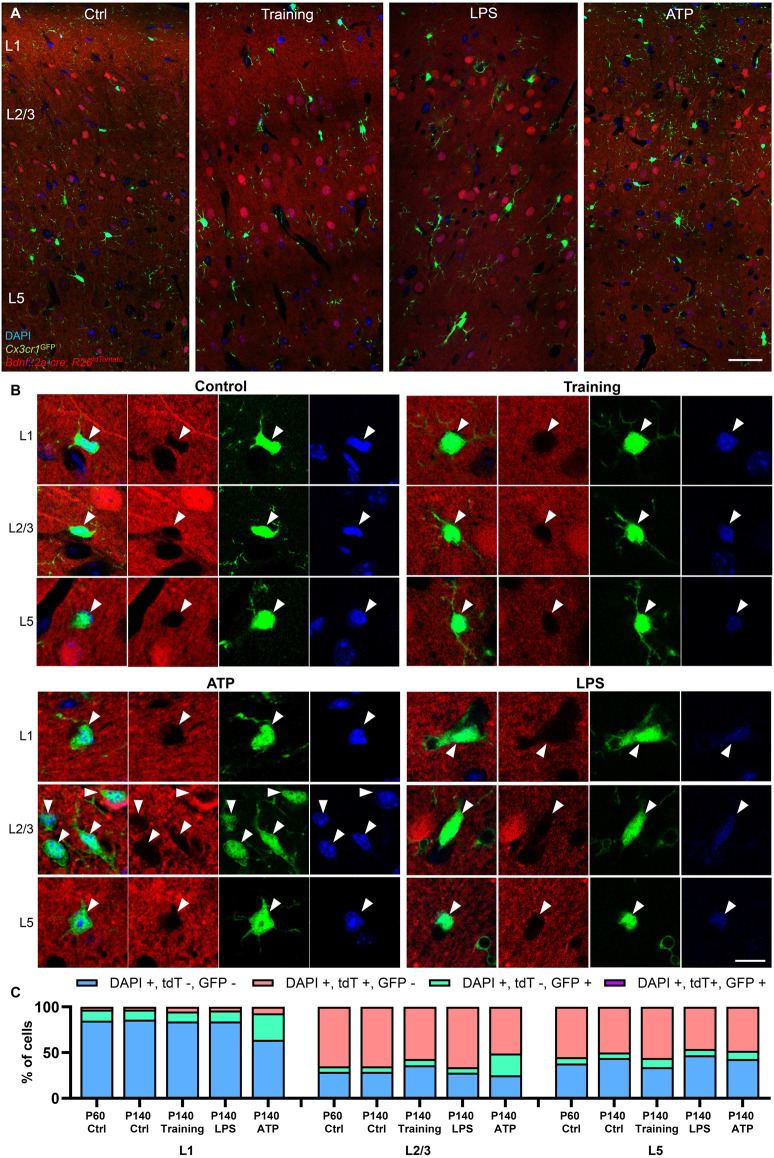
Training and proinflammatory stimuli do not induce BDNF expression in microglia. **(A)** Representative images of the motor cortex at P140 in the *Bdnf*::*2a-cre*; *R26*^LSL-tdTomato^; *Cx3cr1*^GFP^ reporter system in control conditions, after motor training, 2 days after LPS stimulation, and 7 days after ATP stimulation. **(B)** Representative high magnification images of GFP-labeled microglia from L1, L2/3, and L5 showing the absence of tdTomato signal after stimulation. **(C)** Cell quantifications across regions and treatments, showing an increase in GFP-positive microglia after ATP treatment, but no expression of the BDNF reporter in any of the conditions tested. Scale bar: **(A)**: 50 μm, **(B)**: 10 μm.

It is possible that microglia could transiently express BDNF under specific conditions. Because microglia are long-lived cells (Tay et al., 2017), and the constitutive *Bdnf::2a-cre* line crossed with *R26*^LSL-tdTomato^ functions as a cumulative reporter (microglia upregulating *Bdnf* would start expressing tdTomato, and tdTomato-positive microglia would accumulate overtime), we extended our analysis to P140 animals in order to increase the odds of observing recombined microglia (Figures 6A–C). Again, microglia expressing tdTomato were not detected at P140 (*n* = 54 cells analyzed in the motor cortex from two animals at P140).

Microglial BDNF was previously reported to mediate motor training-induced spine formation (Parkhurst et al., 2013). We, therefore, tested the possibility that motor training could upregulate BDNF expression in microglia in the motor cortex, similarly to what has been described in neurons (Andreska et al., 2020). After placing a wheel in their home cage, mice were trained under experimenter control for 4 h per day for three consecutive days. After training, none of the microglia analyzed in the motor cortex expressed tdTomato (*n* = 38 microglia analyzed from two animals at P140, Figures 6A–C).

ATP stimulation was previously reported to upregulate microglial BDNF *in vitro* (Coull et al., 2005; Trang et al., 2009). We attempted to induce BDNF expression in microglia by injecting ATP in the motor cortex of P140 mice. A week after ATP injection, microglia in L1, L2/3, and to a lesser extent L5 demonstrated signs of activation with soma enlargement, processes retraction, and a significant increase in the number of cells per area (Figures 6A–C). However, no microglia were positive for the BDNF reporter (*n* = 207 microglia analyzed around the injection site from two animals).

Because LPS also has been reported to induce BDNF expression in cultured microglia (Prowse and Hayley, 2021), we repeated the same experiment by injecting LPS in the motor cortex. Two days after injection, microglia displayed numerous phagocytic cups (Figure 6B), demonstrating their activation. However, no GFP-positive microglia was observed co-localizing with tdTomato (*n* = 26 microglia analyzed around injection site from two animals at P140), indicating that LPS treatment did not induce BDNF expression in microglia.

Collectively, our results indicate that microglia do not express BDNF, whether in resting or post-training physiological conditions, or upon treatment with proinflammatory stimuli such as ATP and LPS.

## Discussion

Microglia-derived BDNF has previously been suggested to regulate key aspects of neuronal function. However, recent transcriptomic analyses have revealed very low levels of BDNF expression in microglia. We addressed this controversy by assessing the *in vivo* expression pattern of BDNF as well as testing several aspects of neuronal function in the absence of microglial BDNF. We found that postsynaptic protrusion dynamics, training-evoked neuronal activity, as well as microglia-neuron contacts are unaltered in mice without microglial BDNF. Moreover, using several BDNF reporter strategies, we failed to detect any BDNF expression in microglia whether in physiological resting or post-training conditions, or in pathological conditions after LPS or ATP treatments. Our results indicate that BDNF expression in microglia is either absent or too low to detectably modulate neuronal function.

Microglia are part of the innate immune system which mediates primary responses to infection and inflammation by engulfing pathogens and dying cells, as well as releasing pro/anti-inflammatory soluble factors. Hints that microglia could interact with neurons first came from live-imaging studies showing that microglia are highly motile in the normal brain and make transient contacts with dendrites (Davalos et al., 2005; Wake et al., 2009; Tremblay et al., 2010). In the past decade, there has been extensive research investigating microglia’s role in regulating neurogenesis (Sellner et al., 2016; Rodríguez-Iglesias et al., 2019), structural plasticity (Miyamoto et al., 2016; Sipe et al., 2016; Weinhard et al., 2018; Nguyen et al., 2020), and neuronal activity (Badimon et al., 2020; Cserép et al., 2020; Merlini et al., 2021)—all aspects of neuronal function that are also regulated by BDNF. To elucidate the biological function of microglia-derived BDNF, we assessed the number of microglial contacts on neuronal dendrites and their dynamics. We found that *Bdnf* deletion in microglia does not significantly alter microglia-neuron contacts, suggesting that microglia-derived BDNF does not mediate microglia attraction toward neurons. This is in line with the work of Parkhurst et al. (2013) who found no difference in the number of microglial processes in proximity to the dendritic spines using the same mouse model.

In a model of mechanical allodynia, microglial *Bdnf* deletion was shown to block somatosensory cortex hyperactivity induced by nerve injury (Huang et al., 2021). This suggests that microglia can modulate neuronal activity *via* BDNF. We did not observe any significant change in motor training-evoked neuronal activity in the motor cortex in absence of microglial BDNF, although we did find a trend for lower spike frequency. This discrepancy could potentially be explained by differences in the neuronal compartment studied (soma vs. dendrites), the brain region analyzed (somatosensory vs. motor cortex), or the physiological context (injury vs. homeostasis).

In the motor cortex, microglia-derived BDNF was shown to be important for spine formation over 2 days of motor training (Parkhurst et al., 2013). Variation in the net number of spines added in the course of several days can result from changes in either protrusion formation or stabilization which accompany synapse formation. We decided to focus on the analysis of protrusion formation, which represents the initial stage of synapse formation. We did not observe any effect of training on protrusion formation immediately after the session, confirming previous reports suggesting that neuronal activity has a protracted effect on spine formation (Yang et al., 2014). We also found that microglial *Bdnf* deletion did not affect protrusion formation at baseline, or immediately after training. Theoretically, it is possible that microglial BDNF promotes the stabilization of protrusions rather than inducing their formation, or that microglia-derived BDNF promotes spine formation very locally so that some time would be needed after training to readily observe an additive effect on spine density. However, because homeostatic spine formation over the course of weeks was found to be unaffected by the deletion of microglial *Bdnf* (Huang et al., 2021), this is unlikely to be the case.

Microglia were first reported to express BDNF in culture (Elkabes et al., 1996; Batchelor et al., 1999). *In vivo*, microglial expression of BDNF was reported by qPCR from FACS sorted cells (Coull et al., 2005; Parkhurst et al., 2013), and colocalization of *Bdnf* mRNA transcripts with microglia was shown using RNA scope (Huang et al., 2021; Zhang et al., 2021). Using *Bdnf-P2a-Gfp* and *Bdnf::2a-cre*; *R26*^LSL-tdTomato^ reporter systems, we observed that numerous cells express BDNF but no microglia were found to be positive for any of these BDNF reporters (Figures 4–6). This suggests that the level of BDNF expression in microglia is too low to allow GFP expression or cre-mediated tdTomato expression. These data are in line with a recent study showing no expression of BDNF in spinal cord microglia using the same *Bdnf::2a-cre*; *R26*^LSL-tdTomato^ reporter system (Dembo et al., 2018), and with several transcriptomic datasets from microglia in physiological and pathological conditions, showing very low/noise levels of BDNF expression in microglia (Zhang et al., 2014; Bennett et al., 2016; Denk et al., 2016; Ayata et al., 2018; Kang et al., 2018). Interestingly, one transcriptomic analysis of cerebral microglia reported the *Bdnf* gene to be highly enriched for the Trimethylation of histone H3 at lysine 27 (H3K27me3; Ayata et al., 2018), suggesting the *Bdnf* gene is silenced in microglia. This does not rule out the possibility that BDNF could be transiently expressed at low levels, and upregulated under specific conditions. However, even at later timepoints (P140) in resting conditions or after motor training, and after LPS or ATP treatment, no expression of the BDNF reporter was observed in microglia (Figure 6), indicating that BDNF expression was too low in all the conditions tested to induce tdTomato expression. Altogether, one might reasonably question whether such a low level of BDNF expression in microglia, if any, could translate into a biological function, especially in regard to neurons which strongly express BDNF. It has been hypothesized that microglia could slowly accumulate small amounts of BDNF into vesicles, ready to be released in a spatially restricted manner. However, a recent study investigating local translation in distal microglial processes failed to find any *Bdnf* mRNA transcript bound to ribosomes in microglial processes (Vasek et al., 2021), indicating that this possibility is unlikely.

Most of the studies that reported a biological role for microglial BNDF *in vivo* have utilized the same floxed *Bdnf* mouse strain. It is possible that the insertion of loxP sites may alter *Bdnf* accessibility and expression in cells other than microglia, such as neurons, in a cre-independent manner. Although we did not observe any strong phenotype in this floxed *Bdnf* model using our parameters, we did see a trend for lower spike frequency in *Bdnf* cKO mice. More experiments would be needed to verify that the engineered floxed *Bdnf* mouse line does not affect BDNF expression and function in neurons.

Further improvement in the methods for detecting BDNF transcripts might help resolve the controversy on microglial BDNF expression. In particular, it seems crucial to restrict the analysis of microglial BDNF expression to *in vivo* models, since microglial cell lines and primary microglia cultures may be transcriptionally different from *in vivo* microglia. Regarding qPCR detection of *Bdnf* transcripts in FACS sorted microglia from *Cx3cr1*^creER-YFP^ mice, attention should be paid to the purity of these preparations, especially considering that we noticed a few cells other than microglia that expressed low levels of CX3CR1 and were positive for the BDNF reporter in the hippocampus (Supplementary Figures 3C,D). Lastly, combining RNAscope methods with more resolved fluorescence microscopy techniques such as 3D STED may provide better clarity regarding the colocalization of *Bdnf*-RNAscope probes with microglia.

In conclusion, our results suggest that microglia do not express BDNF in sufficient levels to modulate neuronal function. Improvement in the methods for BDNF detection as well as additional transcriptional characterization of the commonly used floxed *Bdnf* mouse model might help further resolve the controversy in the field.

## Data Availability Statement

The original contributions presented in the study are included in the article/Supplementary material, further inquiries can be directed to the corresponding author.

## Ethics Statement

The animal study was reviewed and approved by IACUC.

## Author Contributions

LW designed and performed all the experiments and DH performed all the analysis, except for the TMEM119/NeuN staining in *Bdnf-P2a-Gfp* mouse which was performed and analyzed by EW. LW and EK wrote the manuscript. All authors contributed to the article and approved the submitted version.

## Supplementary Material

Supplementary Figure 1 Tamoxifen treatment efficiently drives recombination. Representative examples of microglia co-labeled with YFP and tdTomato in *Cx3cr1*^*creER-YFP*^; *R26*^*LSL-tdTomato*^ mice. Scale bar: 20 μm.

Supplementary Figure 2 Training does not induce protrusion formation in the hour following training. **(A)** Paradigm to assess protrusion formation in resting state and after training using two-photon *in vivo* imaging of *Thy1*-YFP in L1 of the motor cortex. **(B)** Motor training does not have an effect on protrusion formation in the first hour after training (*n* = 49 dendrites from seven animals in each group, *p* = 0.82, two-way ANOVA). No genotype effect was observed (*p* = 0.96, two-way ANOVA). Scale bar: 5 μm.

Supplementary Figure 3 Non-microglial cells expressing the tdTomato BDNF reporter. Representative examples of **(A)** tdTomato+, GFP- astrocyte-like cell in L1, **(B)** tdTomato+ GFP- vessel-lining cells in L1, **(C)** tdTomato+, GFP+ vessel-associated cells in SR, **(D)** tdTomato+, GFP+ neurons in the SP. Scale bar: 20 μm.

Supplementary Figure 4 Microglia do not express BDNF in the hippocampus. Hippocampal section showing the absence of tdTomato expression in GFP-labeled microglia in the *Stratum Oriens* (*SO*), *Stratum Pyramidale* (*SP*), and *Stratum Radiatum (SR)*. Scale bar: 10 μm.

Supplementary Figure 5 The microglia-specific *Tmem119*^creER^ line induces strong expression of the tdTomato reporter in microglia. Representative 3D views of microglia from *Tmem119*^*creER*^ crossed with *R26*^*LSL-tdTomato*^. Scale bar: 10 μm.
