# Supplementary figures and images for: Analysis of microglial BDNF function and expression in the motor cortex
Source: Front Cell Neurosci. 2022 Dec 23;16:961276. doi: 10.3389/fncel.2022.961276 (PMC9885322; doi:10.3389/fncel.2022.961276)

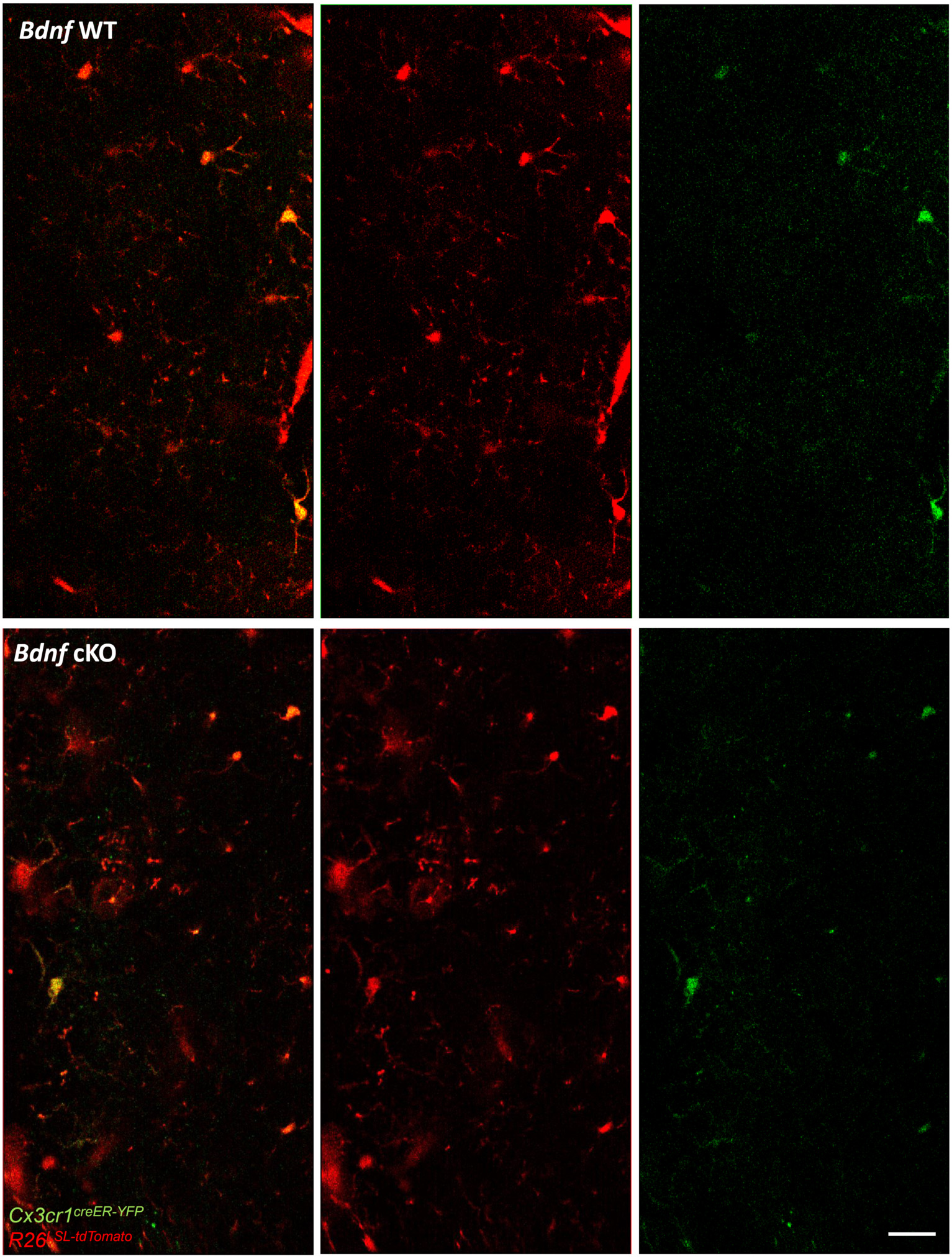

Supplement: Supplementary Figure 1 — Tamoxifen treatment efficiently drives recombination. Representative examples of microglia co-labeled with YFP and tdTomato in Cx3cr1creER-YFP; R26LSL-tdTomato mice. Scale bar: 20 μm. [file Image_1.jpg]

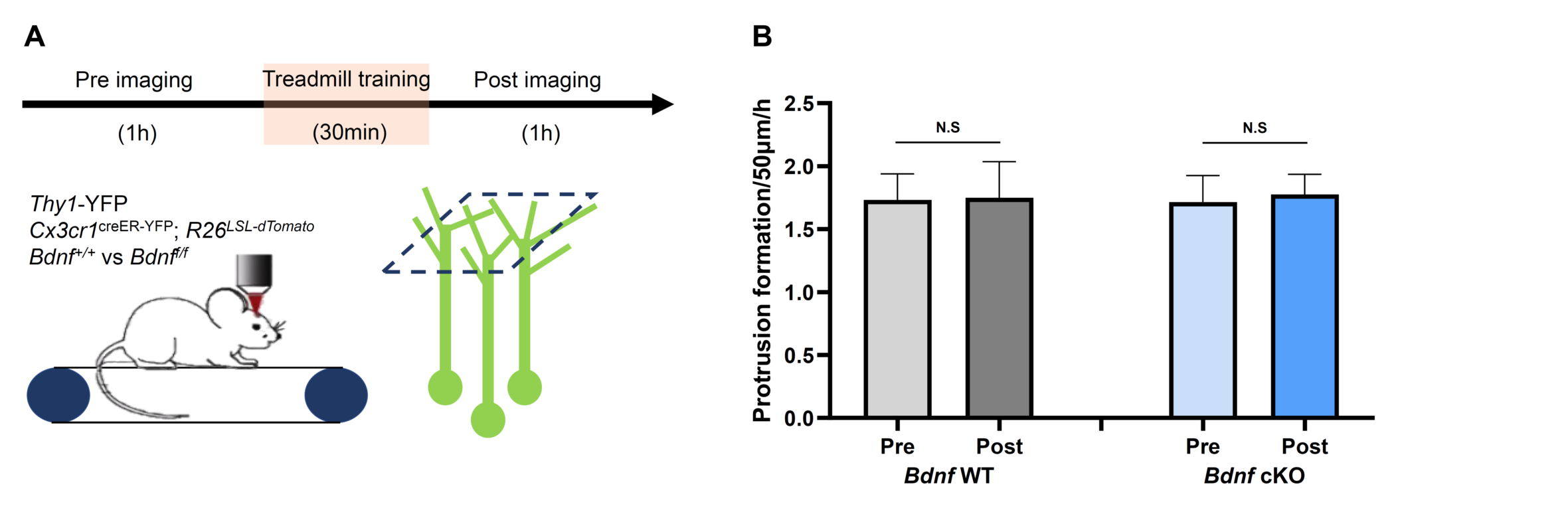

Supplement: Supplementary Figure 2 — Training does not induce protrusion formation in the hour following training. (A) Paradigm to assess protrusion formation in resting state and after training using two-photon in vivo imaging of Thy1-YFP in L1 of the motor cortex. (B) Motor training does not have an effect on protrusion formation in the first hour after training (n = 49 dendrites from seven animals in each group, p = 0.82, two-way ANOVA). No genotype effect was observed (p = 0.96, two-way ANOVA). Scale bar: 5 μm. [file Image_2.tif]

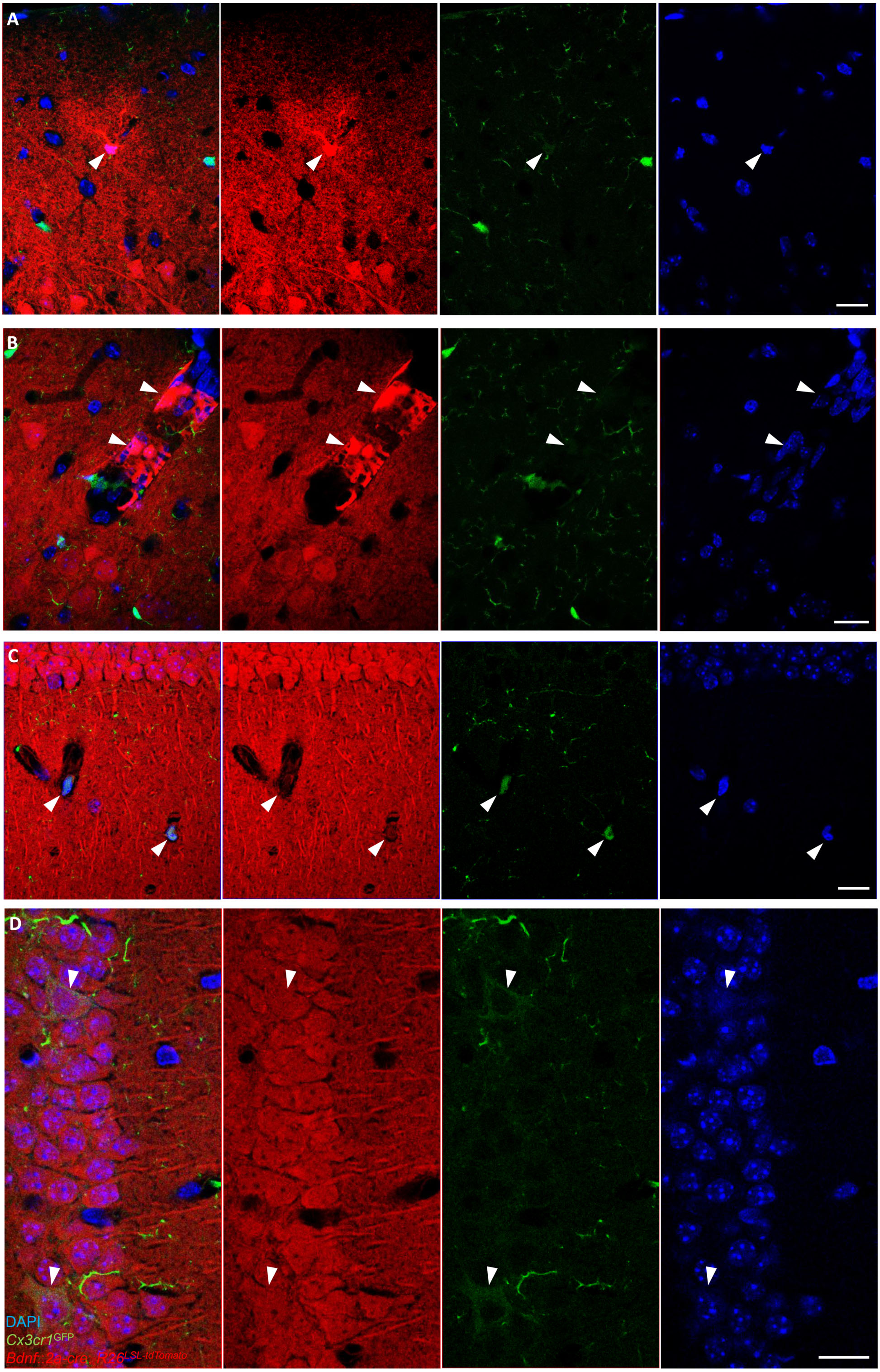

Supplement: Supplementary Figure 3 — Non-microglial cells expressing the tdTomato BDNF reporter. Representative examples of (A) tdTomato+, GFP- astrocyte-like cell in L1, (B) tdTomato+ GFP- vessel-lining cells in L1, (C) tdTomato+, GFP+ vessel-associated cells in SR, (D) tdTomato+, GFP+ neurons in the SP. Scale bar: 20 μm. [file Image_3.jpg]

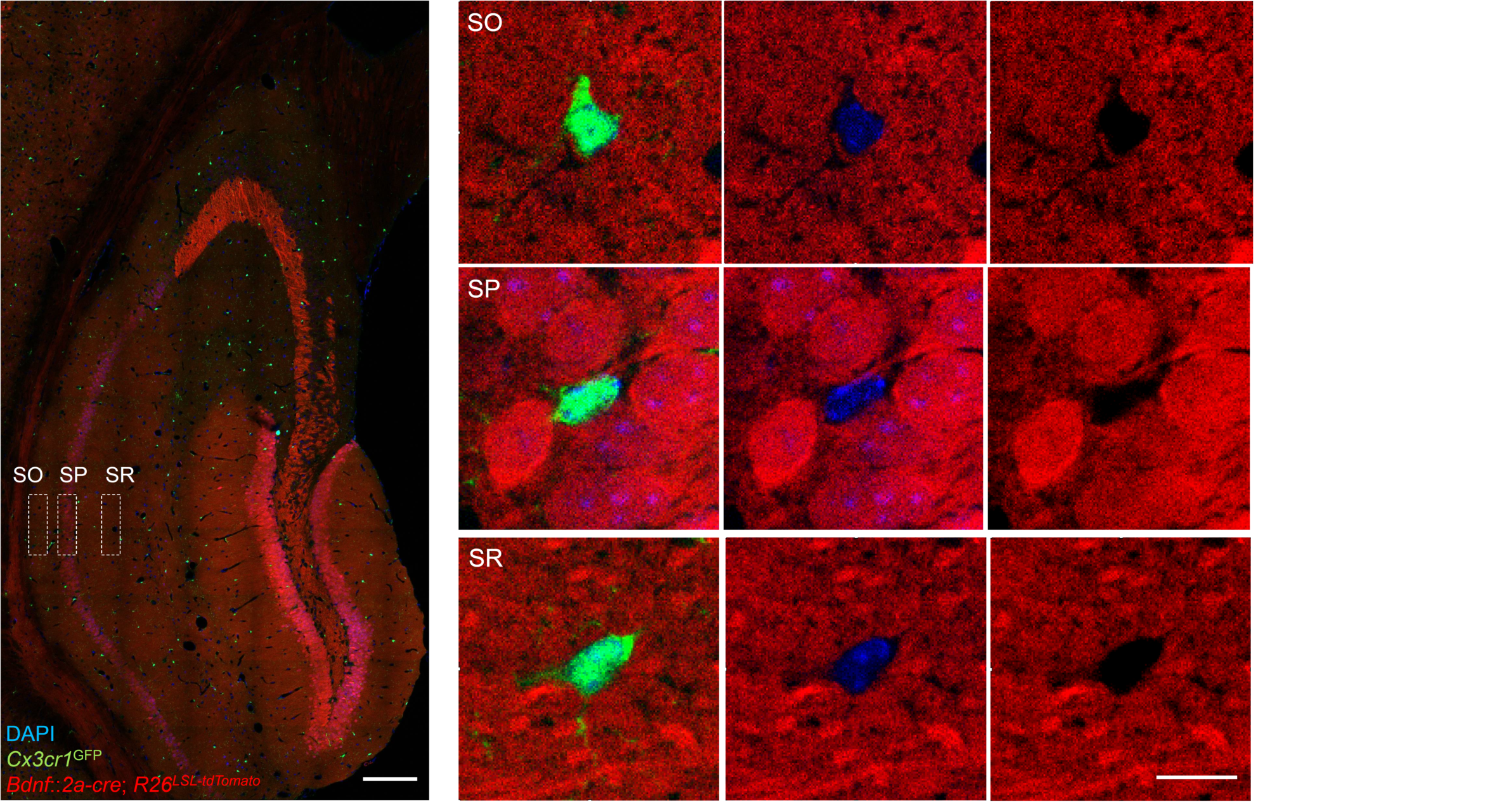

Supplement: Supplementary Figure 4 — Microglia do not express BDNF in the hippocampus. Hippocampal section showing the absence of tdTomato expression in GFP-labeled microglia in the Stratum Oriens (SO), Stratum Pyramidale (SP), and Stratum Radiatum (SR). Scale bar: 10 μm. [file Image_4.tif]

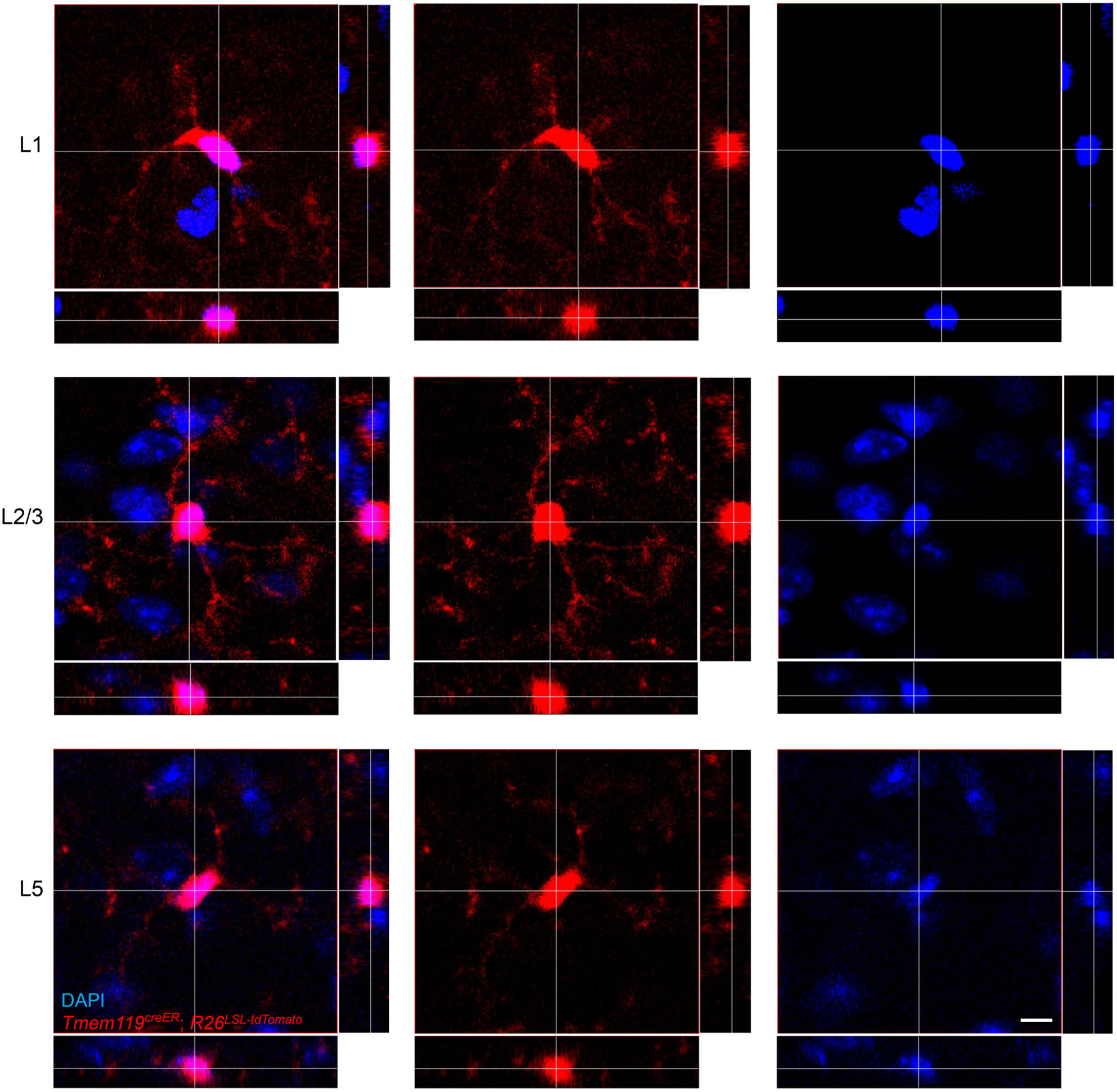

Supplement: Supplementary Figure 5 — The microglia-specific Tmem119creER line induces strong expression of the tdTomato reporter in microglia. Representative 3D views of microglia from Tmem119creER crossed with R26LSL-tdTomato. Scale bar: 10 μm. [file Image_5.jpg]
